# Supplementary material for: Splice-Junction-Based Mapping of Alternative Isoforms in the Human Proteome
Source: Cell Rep. Author manuscript; Available in PMC 2020 Jan 15. (PMC6961840; doi:10.1016/j.celrep.2019.11.026)
Supplement: 2 [file NIHMS1546469-supplement-2.zip › DF1/PXD006675/AorticValve/AorticValve_4_PHACTR4_YHRPQPTTEPGMVLDSVEAGDTTPPTK.pdf]

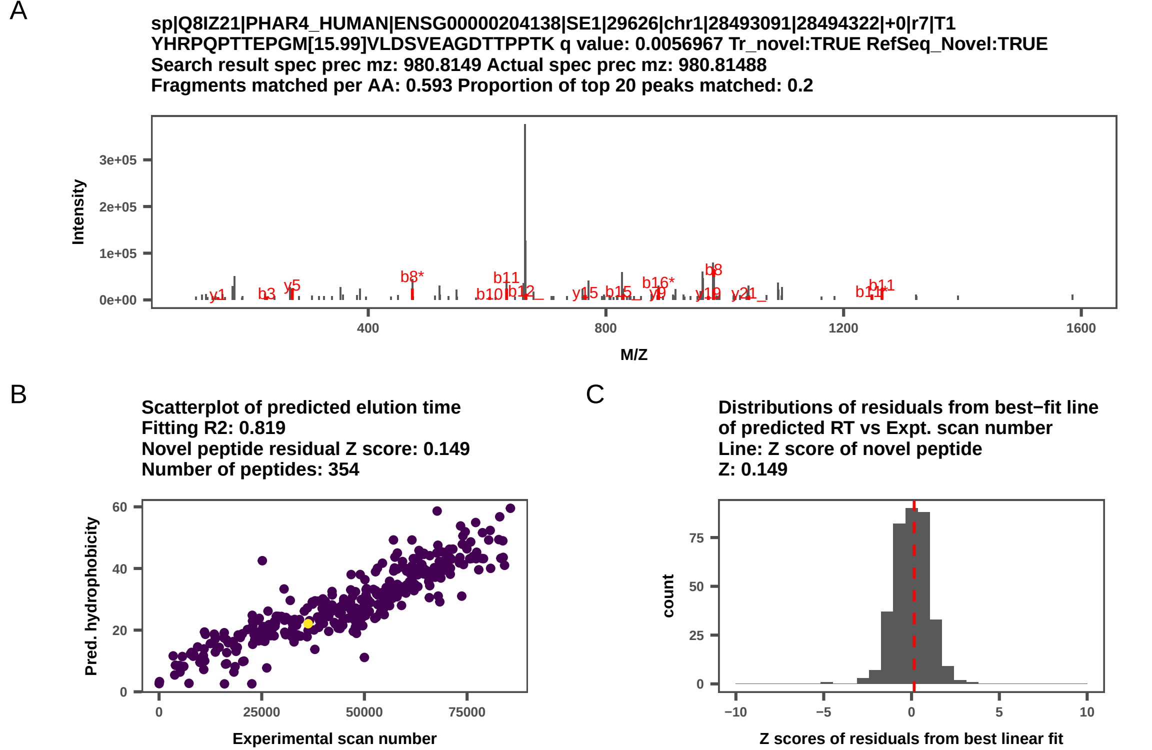
A
sp|Q8IZ21|PHAR4_HUMAN|ENSG00000204138|SE1|29626|chr1|28493091|28494322|+0|r7|T1
YHRPQPTTEPGM[15.99]VLDSVEAGDTTPPTK q value: 0.0056967 Tr_novel:TRUE RefSeq_Novel:TRUE
Search result spec prec mz: 980.8149 Actual spec prec mz: 980.81488
Fragments matched per AA: 0.593 Proportion of top 20 peaks matched: 0.2
Intensity
3e+05
2e+05
1e+05
0e+00
y1
b3
y5
b8*
b10
b11
b12
y15
b15
b16*
y9
b8
y19
y21
b11*
b11
400
800
1200
1600
M/Z
B
Scatterplot of predicted elution time
Fitting R2: 0.819
Novel peptide residual Z score: 0.149
Number of peptides: 354
Pred. hydrophobicity
60
40
20
0
0
25000
50000
75000
Experimental scan number
C
Distributions of residuals from best−fit line
of predicted RT vs Expt. scan number
Line: Z score of novel peptide
Z: 0.149
count
75
50
25
0
−10
−5
0
5
10
Z scores of residuals from best linear fit
